# Epidemic Hepatitis

**Published:** 1847-03

**Authors:** John G. House

**Affiliations:** Springville, N. Y.


*Epidemic Hepatitis*, by John G. House, M. D.

*Springville, N. Y., February* 8, 1847.

Dr. Flint :

In looking over the Boston Medical and Surgical Journal of January 13th, 1847, I find an article entitled Epidemic Hepatitis, in which the Author describes a disease so exactly resembling one which has prevailed in this place during the last three months, that I am induced to give a short description of its history and pathology. My attention was first directed to the subject in November, by being called to attend on four cases of intense Icterus in the space of ten days. I had not previously seen a case of the kind in several months. They were ordinary cases of Jaundice, which uniformly yielded to three or four small powders of calomel, followed by a cathatic of the Black dose, (Salts and Senna) or Ol Ricini. The advance of the disease in these cases was quite mild—consequently I was not called in till the color had become very intense. But the remaining five cases were of a severer grade. They were ushered in by chills and severe pain in the epigastrium towards the right Hypochondrium, of a spasmodic character, causing the patient to cry out. There was most exquisite tenderness on pressure, not only over the epigastrium, but the whole of the bowels. In most cases the bowels were not obstinately constipated, but were usually moved by a large dose of

Calomel and Castor Oil. The pain and spasm were extremely obstinate, often continuing three or four days, notwithstanding recourse to copious venesections, warm bath and opiates, in large doses. Epispastics over the right Hypochondrium had a very good effect in all the cases. Relapses were frequent, and convalescence slow. Finally, a slight mercurial impression seemed necessary to eradicate the disease. The pathology of this disease I believe to be inflammation of the mucus coat of the duodenum extending up the ductus communis to the concave surface of the liver, in some cases producing spasm of the duct and Icterus. I saw no cases complicated with Typhus—yet Typhoid Fever was prevalent at that time.*

Yours truly,

JOHN G. HOUSE.

---

